# Mutation of a nicotinic acetylcholine receptor β subunit is associated with resistance to neonicotinoid insecticides in the aphid *Myzus persicae*

**DOI:** 10.1186/1471-2202-12-51

**Published:** 2011-05-31

**Authors:** Chris Bass, Alin M Puinean, Melanie Andrews, Penny Cutler, Miriam Daniels, Jan Elias, Verity Laura Paul, Andrew J Crossthwaite, Ian Denholm, Linda M Field, Stephen P Foster, Rob Lind, Martin S Williamson, Russell Slater

**Affiliations:** 1 Centre for Sustainable Pest and Disease Management, Rothamsted Research, Harpenden, AL5 2JQ, UK; 2 Syngenta Crop Protection, Werk Stein, Schaffhauserstrasse, Stein, CH4332, Switzerland; 3 Syngenta Crop Protection, Jealotts Hill Intl. Research Centre, Bracknell, RG42 6EY, UK

## Abstract

**Background:**

*Myzus persicae *is a globally important aphid pest with a history of developing resistance to insecticides. Unusually, neonicotinoids have remained highly effective as control agents despite nearly two decades of steadily increasing use. In this study, a clone of *M. persicae *collected from southern France was found, for the first time, to exhibit sufficiently strong resistance to result in loss of the field effectiveness of neonicotinoids.

**Results:**

Bioassays, metabolism and gene expression studies implied the presence of two resistance mechanisms in the resistant clone, one based on enhanced detoxification by cytochrome P450 monooxygenases, and another unaffected by a synergist that inhibits detoxifying enzymes. Binding of radiolabeled imidacloprid (a neonicotinoid) to whole body membrane preparations showed that the high affinity [3H]-imidacloprid binding site present in susceptible *M. persicae *is lost in the resistant clone and the remaining lower affinity site is altered compared to susceptible clones. This confers a significant overall reduction in binding affinity to the neonicotinoid target: the nicotinic acetylcholine receptor (nAChR). Comparison of the nucleotide sequence of six nAChR subunit (Mpα1-5 and Mpβ1) genes from resistant and susceptible aphid clones revealed a single point mutation in the loop D region of the nAChR β1 subunit of the resistant clone, causing an arginine to threonine substitution (R81T).

**Conclusion:**

Previous studies have shown that the amino acid at this position within loop D is a key determinant of neonicotinoid binding to nAChRs and this amino acid change confers a vertebrate-like character to the insect nAChR receptor and results in reduced sensitivity to neonicotinoids. The discovery of the mutation at this position and its association with the reduced affinity of the nAChR for imidacloprid is the first example of field-evolved target-site resistance to neonicotinoid insecticides and also provides further validation of exisiting models of neonicotinoid binding and selectivity for insect nAChRs.

## Background

The peach-potato aphid, *Myzus persicae *is a globally important pest of a broad range of arable and horticultural crops principally due to its ability to transmit more than 100 plant viruses [1]. Its control relies almost exclusively on the application of insecticides and, as a result, this species has developed multiple resistance to many chemical classes including organophosphates, carbamates and pyrethroids [2]. The molecular mechanisms of resistance to insecticides in *M. persicae *include overproduction of detoxifying carboxylesterases (E4 or FE4) which confers resistance primarily to organophosphates, and two forms of target-site resistance involving mutation of the acetylcholinesterase protein, (modified acetylcholinesterase, MACE) giving insensitivity to dimethyl carbamates, and of the voltage-gated sodium channel (knockdown resistance, kdr) giving resistance to pyrethroids [2]. Neonicotinoids such as imidacloprid, thiamethoxam, clothianidin and acetamiprid are unaffected by these mechanisms and are currently the main means of control. Despite two decades of steadily increasing use neonicotinoids have proved remarkably resilient to the development of resistance and have remained highly effective against *M. persicae*. Samples of *M. persicae *with reduced susceptibility to neonicotinoid compounds (10-40 fold resistance) have been found in Europe, the USA and Japan [3-5]. However, at present the levels of resistance described have limited practical significance as they are insufficient to impair the field effectiveness of these insecticides [3,5].

Recently, biochemical and genomic approaches were used to investigate neonicotinoid resistance in a *M. persicae *clone from Greece showing ~40 fold resistance to neonicotinoids [6,7]. Resistance was associated with multiple duplications of a single P450 gene (*CYP6CY3*), with resistant aphids carrying ~18 copies of the gene compared to the two copies found in susceptible aphids. However, ligand-binding and sequencing studies provided no evidence that structural modification of the nicotinic acetylcholine receptor (nAChR; the neonicotinoid target site) contributed to resistance in this clone. Although aphids have continued to be effectively controlled by neonicotinoids, resistance is a significant problem in other insect species including the Colorado potato beetle (*Leptinotarsa decemlineata*), the brown planthopper (*Nilaparvata lugens*) and the tobacco whitefly (*Bemisia tabaci*) [5]. As found for *M. persicae*, overexpression of one or more P450s appears to be the primary mechanism of neonicotinoid resistance in insect pests [5,8]. The only alternative mechanism described to date was modification of the target-site in a laboratory-selected strain of *N. lugens*, when resistance was associated with a point mutation in two nAChR alpha subunits (Nlα1 and Nlα3) [9]. However, resistance in field populations of *N. lugens *appears to occur exclusively via P450-mediated detoxification [10,11] and, to date, no case of target-site insensitivity to neonicotinoids has been described in individuals of any insect pest collected directly from the field.

The selectivity of neonicotinoids for insects is thought to be due, at least in part, to their high affinity for insect nAChRs [12]. Nicotinic receptors are ligand-gated ion channels made up of five subunits arranged in combinations from a family of different subunit subtypes [13]. Insect genomes sequenced to date have contained around ten genes encoding alternative nAChR subunit subtypes and six genes have been characterised in *M. persicae *[14].

We report here, for the first time, a clone of *M. persicae *with extremely potent resistance to neonicotinoids that compromises the field effectiveness of members of this insecticide class. We provide evidence that both P450-mediated detoxification of neonicotinoids and target-site insensitivity confer the resistance phenotype. This is the first example of field-evolved target-site resistance to neonicotinoid insecticides.

## Results

### Topical bioassays

A clone of *M. persicae *(FRC) originating from peach orchards in Southern France exhibited potent resistance to imidacloprid and thiamethoxam when compared to a susceptible clone (4106A) in insecticide bioassays using two different methods of topical application (Table 1). Indeed, using a micro-application method (applying insecticide directly to the dorsal surface of individual aphids), resistance was immeasurable due to insufficient mortality at the highest concentration that could be applied. A comparison of the resistance profile of FRC with 5191A (previously the most neonicotinoid-resistant *M. persicae *clone described) using the same bioassay method (Table 1) highlights the significantly enhanced level of resistance exhibited by FRC. Spray application bioassays, which combine direct contact with insecticide and subsequent exposure to residues on leaves, gave measurable resistance factors of 1679- and 225-fold to imidacloprid and thiamethoxam respectively. In the micro-application assays there was evidence that pre-treatment with the metabolic enzyme inhibitor piperonyl butoxide (PBO) synergised the effect of thiamethoxam (Table 1) although high levels of resistance remained (RF of 800). The effect of pre-treatment with PBO was more evident in spray application bioassays, with resistance factors reduced to 234-fold for imidacloprid and 26-fold for thiamethoxam (Table 1).

**Table 1:** Response of *Myzus persicae *clones 4106A (susceptible), 5191A (moderately resistant) and FRC (highly resistant) to imidacloprid and thiamethoxam using two bioassay methods, with and without pretreatment with piperonyl butoxide (PBO)

		Micro-application			Spray application		
**Treatment**	**Clone**	**LC_50 _^1^(mg l^-1^)**	**95%CL**	**RF^2^**	**LC_50 _(mg l^-1^)**	**95% CL**	**RF^2^**

Imidacloprid	4106A	0.99	0.9-1.4		0.14	0.11-0.17	-
	5191A	31.1	7-69.7	31.4	-	-	-
	FRC	28% at 1000 mg l^-1^	n/c	n/c	235.02	142.33-476.29	1679
Imidacloprid+PBO	4106A	0.1	0.07-0.15		0.03	0.01-0.10	-
	5191A	1.55	0.56-2.55	14.9	-	-	-
	FRC	30% at 1000 mg l^-1^	n/c	n/c	7.03	1.77-37.91	234
Thiamethoxam	4106A	0.64	0.54-0.79		0.48	0.42-0.55	-
	5191A	19.7	14.87-25.74	30.8	-	-	-
	FRC	10% at 1000 mg l^-1^	n/c	n/c	107.84	69.52-185.36	225
Thiamethoxam+PBO	4106A	0.27	0.18-0.32		0.19	0.09-0.41	-
	5191A	1.06	0.77-1.45	3.93	-	-	-
	FRC	216	110.25-483.48	800	4.86	2.03-14.91	26

5191A was only tested by micro-application of insecticide.

^1^Calculated concentration to kill 50% of individuals. If unobtainable, % mortality at the highest concentration applied

^2 ^Resistance factor (LC_50 _resistant/LC_50 _susceptible). n/c = not calculable

### Imidacloprid metabolism

The metabolism of imidacloprid (1-(6-chloro-3-pyridylmethyl)-N-nitroimidazolidin-2-ylideneamine) has been well characterised in mammals, plants, soil and some insect species [15]. Whilst a range of primary metabolites have been identified, the major metabolic pathway identified in many species is hydroxylation and desaturation of the imidazolidine ring to form the hydroxy and olefin metabolites [15]. Initial LC-MS/MS analysis investigating ten selected metabolites (Additional file 1) revealed that the 4/5-hydroxy metabolite was the only metabolite detected in aphids treated with a topical application of imidacloprid. Therefore, subsequent analysis focused on parent imidacloprid and the 4/5-hydroxy metabolite to determine whether there were differences in imidacloprid metabolism between FRC and 4106A.

**Figure 1:**
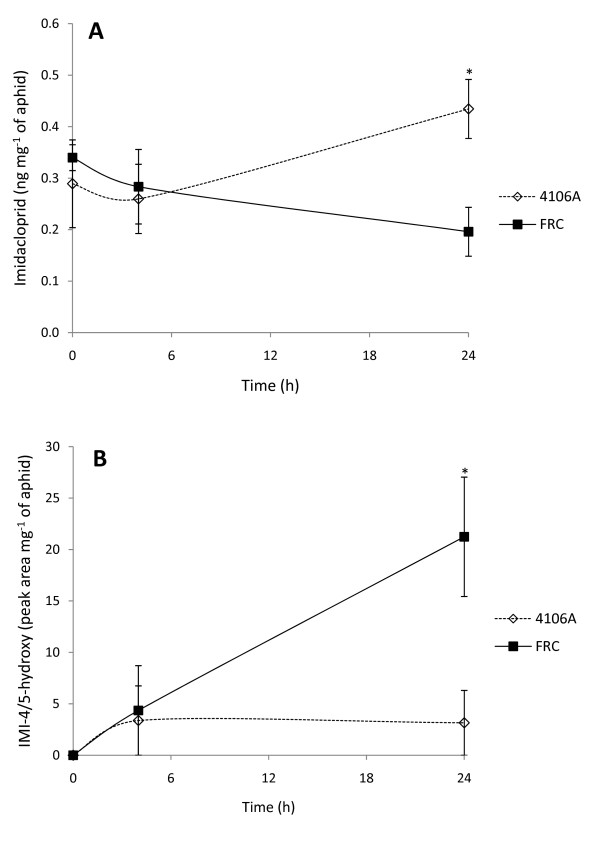
**Internal concentrations of A) imidacloprid and B) 4/5-hydroxy-imidacloprid from aphids treated with a topical application of imidacloprid**. Graphs represent mean recovery of A) imidacloprid (ng mg^-1 ^of aphids) and B) 4/5-hydroxy imidacloprid (relative peak area mg^-1 ^of aphids) ± SE (*n *= 3 pooled samples of 250 aphids). * Denotes a statistically significant difference between 4106A and FRC clones with p < 0.05 (1-tailed Student's *t*-test).

LC-MS/MS analysis did reveal significant differences in imidacloprid metabolism between FRC and 4106A. For 4106A, recovery of imidacloprid increased in a time-dependent manner over the course of the experiment, reaching a maximum of 0.43 (± 0.06) ng mg^-1 ^of aphids after 24 h (Figure 1A), suggesting that the rate of imidacloprid uptake across the cuticle was greater than the rate of imidacloprid metabolism and excretion. In contrast, for FRC the recovery of imidacloprid decreased in a time-dependent manner, reaching a minimum of 0.19 (± 0.05) ng mg^-1 ^of aphids after 24 h (Figure 1A). In terms of metabolite production, in clone 4106A recovery of the 4/5-hydroxy metabolite was variable, often being below the limit of detection and/or limit of quantification. However, in the FRC clone, this metabolite was more consistently detected and was produced in a time-dependant manner, reaching a maximum (21.24 (± 5.80) relative peak area/mg of aphid) after 24 h (see Figure 1B). Although the 4/5-hydroxy metabolite has been shown to possess some insecticidal activity, its specific activity at the nAChR is weaker than imidacloprid [16].

### Microarray Analysis

Microarray analysis identified 571 genes significantly differentially transcribed between FRC and 4106A. The full list of these genes along with Log_2_, calculated fold-change values and a description based on the closest BLAST hit is given in Additional file 2. 349 genes (213 of unknown function) had elevated expression in FRC and 222 (143 of unknown function) were under-transcribed in this clone relative to 4106A. Of the 136 over-expressed genes with a known function, 26 were potential candidates for being involved in insecticide resistance and are listed in Table 2. These included genes encoding cytochrome P450s, carboxylesterase E4/FE4 and a glutathione-S-transferase.

**Table 2:** Selected genes identified by microarray as significantly differentially transcribed between the insecticide resistant *M. persicae *clone (FRC) and the susceptible clone (4106A)

Description	Mean Log2	Fold change	Parent sequence ID
ref|X74555.1| M.persicae mRNA for esterase FE4	8.18	290.98	contig3118
ref|X74555.1| M.persicae mRNA for esterase FE4	6.99	127.91	454Myzus_30192
ref|X74554| M.persicae mRNA for esterase E4	5.95	62.07	contig720
ref|X74555.1| M.persicae mRNA for esterase FE4	5.78	55.25	contig9215
ref|X74554| M.persicae mRNA for esterase E4	5.44	43.60	contig720
ref|X74554| M.persicae mRNA for esterase E4	5.40	42.39	contig4586
ref|HM009309|Myzus persicae cytochrome P450 (CYP6CY3) mRNA, complete cds	3.76	13.60	contig749
ref|HM009309|Myzus persicae cytochrome P450 (CYP6CY3) mRNA, complete cds	3.72	13.26	contig749
ref|HM009309|Myzus persicae cytochrome P450 (CYP6CY3) mRNA, complete cds	3.46	11.01	contig497
ref|HM009309|Myzus persicae cytochrome P450 (CYP6CY3) mRNA, complete cds	3.37	10.40	contig497
ref|HM009309|Myzus persicae cytochrome P450 (CYP6CY3) mRNA, complete cds	3.34	10.18	454Myzus_77428
ref|HM009309|Myzus persicae cytochrome P450 (CYP6CY3) mRNA, complete cds	3.19	9.15	contig5173
ref|XM_001952692.1| PREDICTED: Acyrthosiphon pisum similar to cytochrome P450 (LOC100163313)	2.12	4.36	454Myzus_27444
ref|XM_001946504.1| PREDICTED: Acyrthosiphon pisum similar to glutathione S-transferase-like protein, transcript variant 1 (LOC100168850)	1.55	2.94	contig3513
ref|XM_001947885.1| PREDICTED: Acyrthosiphon pisum similar to cytochrome P450 CYP6AX1 protein (LOC100160895)	1.49	2.83	454Myzus_26873
ref|XP_001944530.1| PREDICTED: similar to cytochrome P450 4A7 [Acyrthosiphon pisum]	1.47	2.78	contig2519
ref|XM_001944991.1| PREDICTED: Acyrthosiphon pisum similar to cytochrome P450 4A7 (LOC100162710)	1.43	2.70	454Myzus_27229
ref|XM_001944991.1| PREDICTED: Acyrthosiphon pisum similar to cytochrome P450 4A7	1.41	2.67	contig1504
ref|XM_001944495.1| PREDICTED: Acyrthosiphon pisum similar to cytochrome P450 4A7 (LOC100158738)	1.40	2.65	454Myzus_82930
ref|XM_001944991.1| PREDICTED: Acyrthosiphon pisum similar to cytochrome P450 4A7	1.30	2.55	contig1504
ref|XM_001947885.1| PREDICTED: Acyrthosiphon pisum similar to cytochrome P450 CYP6AX1 protein (LOC100160895)	1.34	2.55	contig643
ref|XM_001947885.1| PREDICTED: Acyrthosiphon pisum similar to cytochrome P450 CYP6AX1 protein (LOC100160895)	1.33	2.52	DW362118.1
ref|XM_001947885.1| PREDICTED: Acyrthosiphon pisum similar to cytochrome P450 CYP6AX1 protein (LOC100160895)	1.26	2.40	contig643
ref|XM_001948546.1| PREDICTED: Acyrthosiphon pisum similar to cytochrome P450 CYP6AX1 protein	1.23	2.35	454Myzus_74544
ref|XM_001948386.1| PREDICTED: Acyrthosiphon pisum similar to cytochrome P450 CYP6AX1 protein	1.18	2.28	contig4886
ref|NM_001163211.1| Acyrthosiphon pisum cytochrome P450 protein	1.07	2.10	contig1501

Five EST sequences encoding carboxylesterase FE4 and the closely related variant E4 were identified as over-expressed in the resistant clone. However the level of expression was variable (ranging from 42-291-fold) probably because as reported previously only one of the five ESTs (contig 3118) is a perfect match with E4/FE4 [7]. It is likely that the fold-change indicated by hybridisation to the probe designed on contig 3118 is the most accurate and this showed a ~290-fold over-expression.

Five EST sequences (Table 2) corresponding to the *M. persicae *cytochrome P450 gene *CYP6CY3 *showed elevated expression in the FRC clone (9-14-fold). Overexpression of this gene was reported previously to be associated with moderate levels (25-40-fold) of resistance to neonicotinoids in the *M. persicae *clone 5191A (see introduction). Eleven additional EST sequences encoding P450s were also overexpressed in the FRC clone but the changes observed were relatively low (2-4-fold). Analyses of these sequences indicated that the 11 ESTs probably correspond to seven cytochrome P450 genes. Two are most similar in sequence to the *Acyrthosiphon pisum *P450 genes *CYP380C3 *(represented by ESTs 2519 and 82930), and *CYP380C5v1 *(represented by ESTs 27229 and 1504) that belong to the CYP4 clade. The remaining five genes are most similar to the *A. pisum *P450 genes, *CYP6CY2 *(represented by EST 27444), *CYP6CY4 *(represented by EST 74544), *CYP6CY7 *(represented by ESTs 26873, 643 and DW362118), *CYP6CY8 *(represented by EST 1501) and *CYP6CY17 *(represented by EST 4886) belonging to the CYP3 clade. Finally, a single GST gene most similar in sequence to other insect GSTs of the sigma class showed a moderate (~3-fold) increase in expression in FRC compared to 4106A.

Among the 79 genes of known function that were under-transcribed in FRC relative to 4106A, a limited number of detoxification genes were identified (see Additional file 2). These included four sequences encoding GSTs with a negative fold change of -2 to -10, two sequences encoding CYP6-type cytochrome P450s and one sequence encoding a CYP4-type P450 with a fold change of -2 to -3.5.

Real-time quantitative PCR was used to validate microarray results by examining the expression profile of *CYP6CY3 *and the seven additional over-expressed P450 genes. Expression was compared simultaneously between FRC, 4106A and 5191A (Additional file 3). In all cases the increased transcription of the genes was confirmed (apart from the P450 gene encoded by EST 27444 which could not be amplified by PCR despite repeated attempts using several alternative primer pairs). As shown in Additional file 3 the *CYP6CY3 *gene was over-transcribed in FRC 28-fold compared to 4106A but this was not significantly different to the level of expression in 5191A. Overexpression of this gene in 5191A was previously shown to be due, in part, to a 9-fold amplification of the structural gene [7]. Quantitative PCR was therefore used to determine *CYP6CY3 *gene copy number in the FRC clone using genomic DNA as template and normalising data to the *para *gene present in two copies in diploid insect genomes. FRC showed a fold change in copy number of 8.3 +/- 0.52 comparable to that in 5191A. Of the remaining P450 genes, five (two CYP4 and three CYP6-type) were found to be overexpressed in FRC compared to both 4106A and 5191A although the fold-changes observed were low (2-3-fold). The exception was the P450 gene represented by EST 74544 which showed a higher (7-fold) level of overexpression. However, this gene was also overexpressed at a similar level in 5191A suggesting it is not involved in the enhanced resistance phenotype displayed by FRC.

### Radioligand binding assays

**Figure 2:**
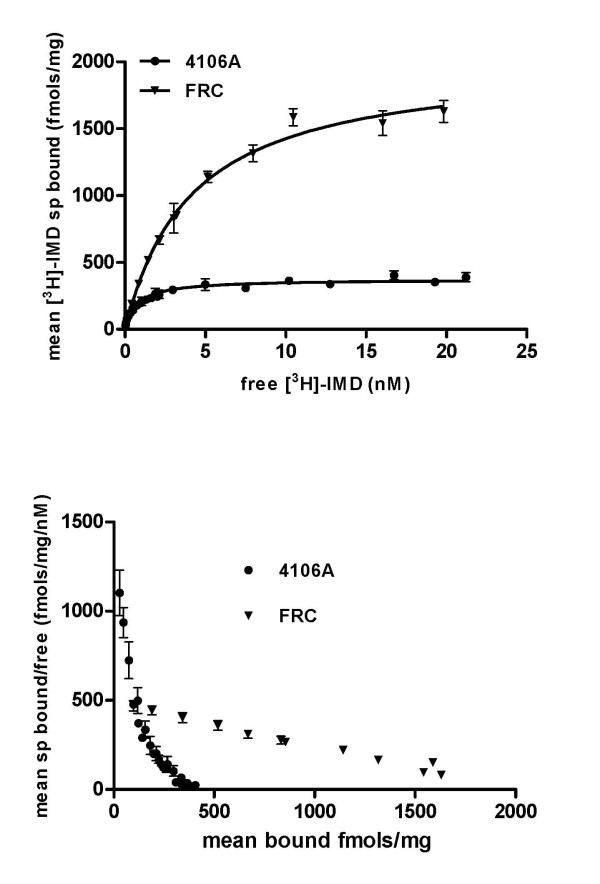
**(A) Comparative saturation isotherms of 3H-IMD binding in crude membranes from 4106A and FRC *Myzus persicae *clones, using MLA as the non-specific ligand (n = 3 +/-SD)**. (B) Scatchard plot of data derived from (A) demonstrating the presence of multiple binding sites in 4106A but only a single binding site in FRC.

Radioligand binding assays of [3H]-imidacloprid to aphid membrane preparations revealed substantial differences in the nature of imidacloprid binding between FRC and 4106A. As described previously, [3H]-imidacloprid recognised two sites in the susceptible 4106A clone, one of very high (sub nM) affinity and a second lower (~nM) affinity site [17] (Figure 2A and 2B, Table 3). In the FRC clone the dual nature of [3H]-imidacloprid binding was absent with only a single lower affinity binding site present, as demonstrated by a Hill value close to unity (Figure 2, Table 3), resulting in an overall reduction in binding affinity. In addition, this single binding site was present at far greater concentrations than that observed for the [3H]-imidacloprid binding sites in 4106A indicated by the substantially increased B_max _value in the FRC clone. The K_d _values recorded in this study for wild-type *M. persicae *differ somewhat from those recorded previously (0.14 ± 0.01 and 12.58 ± 1.83 nM) [17], however, there are some significant experimental differences between the two studies. Firstly, in this study larger final incubation volumes (1 ml) were used to avoid ligand depletion at very low concentrations of added [3H]-imidacloprid. Secondly, two different wild-type strains were used, US1L by Lind et al and 4106A in this study.

**Table 3:** [3H]-imidacloprid saturable binding to membrane preparations of the FRC and 4106A *M. persicae *clones using methyllycaconitine as the non-specific ligand (n = 3 +/-SD)

Clone	High affinity		Low affinity		
	kd (nM)	Bmax (fmols/mg)	kd (nM)	Bmax (fmols/mg)	Hill value (*n*_H_)
FRC	-	-	4.14 (± 0.39)	2016 (± 70.5)	1 (± 0.005)
4106A	0.083 (± 0.0085)	100 (± 55)	1.7 (± 0.65)	298 (± 48)	0.78 (± 0.072)

### Sequence analysis of *M. persicae *nAChR subunit genes

The N-terminal region of the *M. persicae *nAChR α1-α5 and β1 subunits, encompassing the conserved domains (loops A to F) that comprise the acetylcholine and neonicotinoid binding site were amplified by PCR from FRC, 5191A, and 4106A and examined by nucleotide sequencing. Although a limited number of silent SNPs were detected in the nucleotide sequence of the nAChR α1-α5 subunits, no non-synonymous changes were observed and the deduced amino acid sequence in the region studied was identical between all clones. In contrast, when the sequence of the Mpβ1 subunit was compared a non-synonymous SNP was observed exclusive to FRC that causes an amino acid replacement of arginine (AGA) to threonine (ACA) at amino acid position 81 within loop D, a predicted agonist binding site of nAChR β subunits (Table 4). Additional sequencing of RT-PCR products derived from RNA extracted from individuals confirmed that this mutation is homozygous in FRC.

**Table 4:** Alignment of amino acid sequence in loop D of the agonist binding site of vertebrate and insect nicotinic acetylcholine receptors

	Amino Acid Number of *Myzus persicae β1 *Subunit										
Species	**77**	**78**	**79**	**80**	**81**	**82**	**83**	**84**	**85**	**86**	**87**

*Homo sapiens β2*	N	V	W	L	**T**	Q	E	W	E	D	Y
*Gallus gallus β2*	N	V	W	L	**T**	Q	E	W	E	D	Y
*Rattus norvegicus β2*	N	V	W	L	**T**	Q	E	W	E	D	Y
*Drosophila melanogaster β1*	C	V	W	L	**R**	L	V	W	Y	D	Y
*Anopheles gambiae β1*	N	V	W	L	**R**	L	V	W	S	D	Y
*Bemisia tabaci β1*	N	V	W	L	**R**	L	V	W	N	D	Y
*Locusta migratoria β1*	N	V	W	L	**R**	L	V	W	N	D	Y
*Heliothis virescens β1*	N	V	W	L	**R**	L	V	W	M	D	Y
*Ctenocephalides felis β1*	N	V	W	L	**R**	L	V	W	S	D	Y
*Myzus persicae 4106A β1*	N	V	W	L	**R**	L	V	W	R	D	Y
*Myzus persicae 5191A β1*	N	V	W	L	**R**	L	V	W	R	D	Y
*Myzus persicae FRC β1*	N	V	W	L	**T**	L	V	W	R	D	Y

## Discussion

The neonicotinoids are the fastest growing class of insecticides in the global crop protection market with annual worldwide sales of over $1.5 billion [18]. They have remained effective against *M. persicae *because they circumvent the resistance mechanisms that have evolved to other insecticide classes, and have so far not been compromised by the evolution of novel mechanisms. In this study we have characterised a field-derived clone (FRC) of *M. persicae *exhibiting sufficiently high levels of neonicotinoid resistance to impair field performance of these insecticides. The involvement of P450-mediated detoxification in resistance was initially implicated by the use of PBO, an inhibitor of metabolic enzymes including P450s, which was found to alter the phenotype of the resistant clone. Further evidence was provided by LC-MS/MS analysis of imidacloprid metabolism, which demonstrated that the 4/5-hydroxy-imidacloprid metabolite, a product of phase I reactions catalysed by microsomal P450s [15], was produced at higher levels in FRC than the susceptible clone (4106A). Microarray and quantitative real-time PCR analyses showed that a cytochrome P450 gene *CYP6CY3 *was overexpressed in the FRC clone 28-fold and this was due in part to an eight-fold amplification of the structural gene. Amplification of this gene has been reported previously for *M. persicae *where it was associated with moderate (25-40-fold) resistance to neonicotinoids in a clone from Greece (5191A) [7]. However, the levels of expression of this gene in FRC and 5191A were not significantly different and are therefore unlikely to explain the significantly enhanced levels of resistance seen in the FRC clone. A small number of additional P450 genes were found to be overexpressed in FRC compared to both 4106A and 5191A. However, the level of overexpression was relatively low (2-3) fold and while it is possible the proteins encoded by these genes may play some role in resistance it is unlikely their overexpression explains the significantly enhanced level of resistance exhibited by FRC compared to 5191A. The gene encoding carboxylesterase FE4 was also highly overexpressed (~290-fold) in FRC but this is an established mechanism of resistance to other insecticides that has been shown to have no effect on neonicotinoids [7,19].

Bioassays also provided strong evidence of an additional major mechanism(s) in resistance with a significant resistance factor remaining in the FRC clone to both thiamethoxam and imidacloprid after synergism by PBO. To investigate the possibility of target-site insensitivity in resistance, the binding of [3H]-imidacloprid to membrane preparations of FRC and 4106A was compared. Previous binding studies with a range of insect species have demonstrated that while all insect species examined have a binding site for imidacloprid, Hemipteran insects including *M. persicae *have two sites, one of very high affinity and a second of lower affinity [17,20]. This may account for the enhanced efficacy of imidacloprid against sap-feeding insects such as aphids, leafhoppers and planthoppers [17]. In a previous study no difference in the binding of [3H]-imidacloprid to membrane preparations from 5191A and 4106A was observed [7]. However, in this study we found the high affinity [3H]-imidacloprid binding site had been lost in FRC and the remaining lower affinity site was altered, resulting in a substantial overall reduction in binding affinity. Recent study of nAChR subunits in the Hemipteran *N. lugens *has suggested which subunits of the nAChR contribute to the formation of the imidacloprid binding site [17]. This work has shown that the Nlβ1 subunit is an absolute requirement for imidacloprid binding and that nAChRs containing Nlα1, Nlα2 and Nlβ1 constitute the lower affinity site whereas nAChRs containing Nlα3, Nlα8 and Nlβ1 constitute the higher affinity site [20]. To see if mutation of the orthologous nAChR subunits was associated with resistance in FRC the nucleotide sequence of the N-terminal (agonist binding) region of six nAChR subunit (Mpα1-5 and Mpβ1) genes from FRC, 4106A and 5191A was compared. No non-synonymous changes were observed between clones in any nAChR α subunit. However, a single point mutation in the loop D region of the nAChR β1 subunit of the FRC clone was identified that causes an arginine to threonine substitution (R81T). Loop D is one of three regions of the β1 subunit that in combination with loops A, B and C of α subunits form the acetylcholine binding site [21]. Several lines of experimental evidence indicate that the amino acid at this position within loop D is a key determinant of neonicotinoid binding to nAChRs. Insect β1 receptors are highly conserved at this amino acid position and all insect species characterised to date have a positively charged arginine at this position (Table 4). In contrast, vertebrate β subunits rarely have a positively charged amino acid at this position with the most common residue being a threonine (Table 4). Indeed, the high sensitivity of insect nAChRs to neonicotinoids is thought to be due to interactions between the distinctive electronegative pharmacophore (nitro or cyano group) of these insecticides and conserved residues upstream of loop B, within loop C of α subunits, and certain positively charged residues in loop D (such as R81) of β subunits [12,22,23]. Crystallization of molluscan ACh-binding proteins, homologous to the amino-terminal ligand binding domain of nAChR subunits, has confirmed that neonicotinoids bind to nAChRs at the same site as acetylcholine and that amino acids at the corresponding position to R81 are in close proximal contact with imidacloprid [24]. The most compelling evidence that R81T reduces neonicotinoid binding comes from site-directed mutagenesis and homology modelling studies of vertebrate and insect recombinant receptors [22]. Substituting the threonine residue in the chicken β2 subunit at position 77 (corresponding to the Mpβ1 subunit position 81) with arginine or another basic residue greatly enhanced the affinity of recombinant nAChRs (such as *Drosophila melanogaster *Dα2/chicken β2 hybrids) for imidacloprid. However, this effect was only seen following an additional substitution of a nearby residue in the chicken β2 subunit (E79V) to mimic insect β1 subunits which, in contrast to vertebrate β subunits, have a valine at this position. Models of the recombinant receptors indicated that the nitro group of imidacloprid interacts directly with the introduced basic residue at this position [22]. Interestingly, and importantly, the T77R substitution did not significantly affect the binding affinity of the natural agonist acetylcholine. The mutation described in this study therefore appears to confer a 'vertebrate-like' quality to the β1 subunit of resistant aphids resulting in reduced sensitivity of the nAChR to neonicotinoids through the loss of direct electrostatic interactions of the electronegative pharmacophore with the basic arginine residue at this key position within loop D. The discovery of the mutation at a predicted resistance 'hotspot' [22] and its association with the reduced affinity of the nAChR for imidacloprid also provides further validation of existing models of neonicotinoid binding and selectivity for insect nAChRs.

In a recent study of neonicotinoid resistant housefly, *Musca domestica *reduced expression of a nAChR alpha subunit (Mdα2) was correlated with the resistance phenotype [25]. Given that radioligand binding assays revealed significant changes to the nature of imidacloprid binding in the FRC strain it would be interesting, in future, to investigate if changes in the expression of different nAChR subunit genes are also associated with resistance.

The relative role of the two mechanisms (P450-mediated detoxification and target-site insensitivity) in determining the resistance phenotype warrants further study. In particular, the potency and dominance characteristics of target-site resistance alone, and the practical impact of different combinations of mechanisms on the efficacy of foliar and systemic applications of neonicotinoids remain to be investigated. It is possible that target-site resistance has evolved in a genetic background of enhanced P450 production and that the mechanisms act synergistically to confer unprecedented levels of resistance to neonicotinoids in *M. persicae*.

## Conclusions

In summary, we describe, for the first time, a clone of *M. persicae *exhibiting control-compromising levels of resistance to neonicotinoid insecticides. We demonstrate that resistance is associated with enhanced imidacloprid metabolism and over-expression of cytochrome P450s but that additional major mechanisms based on reduced affinity of the target-site to the neonicotinoid imidacloprid are involved. We provide strong evidence that resistance is conferred, in part, through mutation of a key residue in the loop D region of a nAChR β1 subunit and this represents the first example of field-evolved target-site resistance to neonicotinoid insecticides.

## Methods

### Aphid Clones

All aphid lines used in this study were clonal lineages derived from a single founding female. 4106A was an insecticide-susceptible clone originating from potatoes in Scotland in 2000. Clone 5191A originated from tobacco in Greece in 2007, and exhibited 20-40 fold resistance to neonicotinoids [6,7]. Clone FRC was derived from a sample collected from peach orchards in Southern France in 2009, following reports of reduced control with neonicotinoids. All clones were reared on Chinese cabbage under standardised conditions [7].

### Insecticide Bioassays

Two bioassay methods were used to establish the extent of neonicotinoid resistance in the FRC clone using insecticide alone and 5 h after pre-treatment with PBO to investigate the potential contribution to resistance of detoxifying enzymes that are inhibited by this synergist [6]. The 'micro-application' bioassay involved dosing individual aphids on the dorsal surface with 0.25 μl of acetone plus insecticide using a micro-applicator (Burkhard) and measuring mortality 72 h later as previously described [7]. The 'spray application' bioassay involved spraying insecticide onto aphids and foliage inside a Potter-Precision laboratory spray tower (Burkhard) [26] For this formulated imidacloprid (Confidor 200 g/L SL Bayer S.A. Division Agro, France) and thiamethoxam (Actara 25WG Syngenta Crop Protection AG, Basel, Switzerland) was used. For synergism assays piperonyl butoxide was formulated by Syngenta chemists as an 80% EC and applied in 0.01% non-ionic surfactant, Extravon (Syngenta Crop Protection AG, Basel, Switzerland). Test pots (45 mm diameter) were prepared with discs of Chinese cabbage on tap water agar. Twenty to thirty mixed age aphids were transferred to the dishes and allowed to settle for 24 h at 21°C with a 16:8 h light regime. Serial dilutions of insecticide were applied in 3 ml of solution at 0.6 bar with 3 second settling time (equivalent to approximately 400L/ha). Three replicates were used for each concentration tested and the responses were assessed after 72 h. Aphids that were dead or seriously affected were classed as 'dead' and LC_50 _values were calculated by LOGIT analysis using Syngenta software.

### Imidacloprid Metabolism Assay

Imidacloprid metabolism assays were carried out to determine which imidacloprid metabolites were observed in aphids and whether increased metabolism was contributing to resistance in the FRC clone. In both experiments, individual aphids of the 4106A and FRC clones were treated by topical application of imidacloprid (0.2 μL, 1 mg/L, corresponding to the LC50 value for 4106A) using a micro-applicator as described in [7]. Owing to the physical constraints of performing topical applications to aphids, aphids were treated in batches, which resulted in a 15 minute gap between the first and last aphid being treated from each batch.

To determine which metabolites were observed in aphids, ~500 treated aphids of each clone were collected at 0, 1, 4 and 24 h after treatment, weighed and frozen at -20°C. Due to the low treatment rate and volume used in this study, 500 aphids were pooled to provide one sample for each time point. To remove surface imidacloprid, aphid samples were washed in acetonitrile (3 × 1 mL), after which, aphid samples were placed in extraction tubes (Precellys 24, 0.5 mL, Lysing matrix VK05; Bertin Technologies, France) containing acetonitrile (0.5 mL). Samples were macerated for 5 × 20 s using a FastPrep^® ^FP120 (QBiogene, UK), centrifuged (5590*g*, 15 min) and the resulting supernatant was transferred into HPLC vials for LC-MS/MS analysis. Separation was achieved with a Accela UPLC pump (Thermo Scientific, UK) using a Phenomoenex Luna (C18, 5 μm, 4.60 × 250 mm) analytical column, with a mobile phase consisting of water (+0.2 % formic acid), and a flow rate of 0.8 mL/min. The gradient elution conditions of acetonitrile:water were as follows: 0 min 10:90, 15 min 47.5:52.5, 15.1 min 80:20, 22 min 80:20, 22.1 min 10:90. The mass spectrometer used was a TSQ Vantage (Thermo Scientific) equipped with a H-ESI II source operating in positive ion mode. Analytes were detected simultaneously using selected-reaction-monitoring (SRM). SRM transitions are outlined in Additional file 1. Scan time was 0.1 s/transition and the collision gas pressure was 1.5 mTorr. As noted in Additional file 1, several metabolites were available as authentic standards. For these metabolites, extraction efficiencies were determined, by spiking untreated aphid samples with a known amount (0.05 μg) prior to performing the maceration and centrifugation steps, which were between 87-94%. For 4/5-hydroxy-imidacloprid, only 5-hydroxy-imidacloprid was available as an authentic standard and it was thought to be unlikely that chromatographic separation of the 4- and 5-hydroxy metabolites would have been achieved. Where no authentic standards were available, confirmation was made by comparison of relative retention times to previous studies [27].

In the second experiment, aphids were treated as described above, with the following modifications: 250 treated aphids of each clone were collected at 0, 4 and 24 h after treatment, weighed and frozen at -20°C. Treatments were replicated three times. Aphid samples were washed in acetonitrile (3 × 0.5 mL), placed in extraction tubes (Precellys 24, 0.5 mL, Lysing matrix VK05) containing acetonitrile (0.25 mL) and prepared as described above. Recoveries of imidacloprid and 4/5-hydroxy metabolite were determined using LC-MS/MS. Separation was achieved using Ultra Performance LC^® ^(ACQUITY UPLC-System; Waters, UK) using an ACQUITY UPLC column (HSS T3, 1.8 μm, 100 × 2.1 mm), with a mobile phase consisting of water (+0.2% formic acid), with a flow rate of 0.6 mL/min. The gradient elution conditions of acetonitrile:water were as follows: 0 min 0:100, 0.5 min 0:100, 3.5 min 95:5, 4.5 min 95:5, 4.6 min 0:100, 5.0 min 0:100. The mass spectrometer used was a Finnigan TSQ Quantum Discovery (Thermo Scientific) equipped with an Ion Max source operating in positive ion mode. Imidacloprid and 4/5-hydroxy metabolite were detected simultaneously using SRM.

### Microarray

The microarray (Agilent Technologies) used in this study was designed by the Georg Jander Lab and is based on a previously described array containing probes for > 10, 000 *M. persicae *unigenes produced by Sanger sequencing [28] augmented with an additional 30, 517 probe set designed on EST unigene sequences identified in a 454 sequencing project [29]. Microarray experiments consisted of four biological replicates and for each of these, two hybridisations were done in which the Cy3 and Cy5 labels were swapped between samples for a total of eight hybridisations between resistant and susceptible clones. Arrays were processed, scanned and the raw data normalised as described previously [7]. The TM4 suite of software from the Institute of Genomic Research was used for statistical analysis using the SAM (Significance Analysis of Microarrays) module and applying a False Discovery Rate (FDR) of zero to detect significantly differentially expressed genes [30]. Genes identified by SAM and showing a transcription ratio > 2 fold in either direction were considered to be differentially transcribed between the two clones. Microarray data were submitted to the Gene Expression Omnibus (GEO) database (series no. GSE24629).

### Quantitative RT-PCR

Quantitative RT-PCR was used to validate microarray data by examining the expression profile of selected genes. Primers were designed to amplify a fragment 90-150bp in size and are listed in Additional file 4. PCR reactions were carried out as described previously [7]. Data were analysed using the geometric mean of three selected housekeeping genes (actin, *para *which encodes the voltage gated sodium channel, and *ace*, which encodes acetylcholinesterase) for normalisation according to the strategy described previously [31]. Quantitative PCR was used to determine *CYP6CY3 *gene copy number using genomic DNA as the template as described previously [7].

### Membrane preparation and [^3^H] imidacloprid binding

Membranes from resistant (FRC) and susceptible (4106A) aphid clones were prepared and radioligand binding experiments performed based on previously described methods [17] with the following modifications. Aphids, (~5 g) were homogenized on ice in 16 ml of pre-chilled homogenisation buffer (0.05 M Tris base, pH 7.4 with HCl, 200 μM PMSF) with a motor-driven Ultra Turrax (5 × 20 sec bursts, level 4.5). The homogenate was centrifuged (3,000 g, 30 mins at 4°C) and resulting supernatant filtered through two layers of pre-wetted mira cloth before a final centrifugation (83,000 g, 60 mins at 4°C). The pellet (crude membrane) was re-suspended in chilled freshly prepared binding buffer (0.05 M Tris, 0.12 M NaCl, 100 μM EDTA, pH7.4 HCl), beaded into liquid N_2 _and stored at -80°C. The protein concentration was determined by Bradford assay using BSA as a standard. Comparative saturation isotherms were performed in side-by-side fashion with appropriate time delay. [^3^H]-imidacloprid (1.1 TBq/mmol, purity > 95%, in-house synthesis) and methyllycaconitine (MLA, Sigma) (non-specific ligand) were prepared in binding buffer. Crude aphid membranes were defrosted on ice and diluted to 5 mg/ml in binding buffer containing 0.2% BSA. Incubations were carried out in a final volume of 1 ml to avoid ligand depletion at very low concentrations of added [^3^H]-imidacloprid (returned bound counts were in all cases less than 10% of added). Order of addition was binding buffer, 100 nM MLA or control, 100 μg membranes followed by [^3^H]-imidacloprid at indicated concentrations. Reactions equilibrated over 90 mins at room temperature with constant shaking and were terminated by filtration using a Brandel harvester onto Whatman GF/B filters pre-soaked in cold binding buffer with 0.25% polyethylenimine (no BSA). Filters were rapidly rinsed in a two wash cycle (3 ml) with ice cold binding buffer (no BSA), removed, allowed to air-dry for 12 hrs and soaked (6 hrs) in 10 ml scintillant (Scintsafe Gel, Fisher) prior to counting.

### Sequence analysis of *M. persicae *nAChR subunits

Total RNA was isolated from three pools of five adult aphids from all clones listed above and first strand cDNA synthesised as described previously [7]. PCR amplification was performed using gene-specific oligonucleotide primers designed to the published nAChRα1 (X81887), nAChRα2 (X81888), nAChRα3 (AJ236786), nAChRα4 (AJ236787), nAChRα5 (AJ880084) and nAChRβ1 (AJ251838) as described previously [7]. Amplicons of the expected size were ethanol precipitated and sequenced directly in both directions using the BigDye mix (Applied Biosystems).

## Authors' contributions

CB carried out the genomics research and analyses and drafted the manuscript. AMP carried out bioassays, nAChR sequencing and analyses and helped draft the manuscript. MA, JE and VLP carried out bioassays and associated analyses, MD and RL carried out the LC-MS/MS analyses and helped draft the manuscript, PC and AJC carried out the ligand binding analyses and helped draft the manuscript, ID, LMF, SPF, and MSW, helped direct the study and draft and critically review the manuscript, RS conceived the project and help draft and critically review the manuscript. All authors read and approved the final manuscript.

## Supplementary Material

Additional file 1 **Selected-Reaction-Monitoring transitions for selected imidacloprid metabolites**. Molecular weight, SRM transitions, collision energies and availability of authentic standards are detailed for 11 imidacloprid metabolites. Click here for file

Additional file 2 **ESTs identified by microarray analysis significantly differentially transcribed between the insecticide resistant *M. persicae *clone FRC and the susceptible clone 4106A**. The full list of ESTs along with Log2, calculated fold-change values and a description based on the closest BLAST hit is detailed. Click here for file

Additional file 3 **Fold change in expression of *CYP6CY3 *and additional P450 genes in the *M. persicae *clones FRC and 5191A (compared to the susceptible reference clone 4106A) as determined by quantitative PCR**. For each EST the most similar *Acyrthosiphon pisum *P450 gene is detailed. Click here for file

Additional file 4 **Sequences of primers used in this study**. Primer sequences are listed along with the purpose for which they were used. Click here for file
