# Supplementary material for: Mutation of a nicotinic acetylcholine receptor β subunit is associated with resistance to neonicotinoid insecticides in the aphid Myzus persicae
Source: BMC Neurosci. 2011 May 31;12:51. doi: 10.1186/1471-2202-12-51 (PMC3121619; doi:10.1186/1471-2202-12-51)
Supplement: Additional file 1 — Selected-Reaction-Monitoring transitions for selected imidacloprid metabolites. Molecular weight, SRM transitions, collision energies and availability of authentic standards are detailed for 11 imidacloprid metabolites. [file 1471-2202-12-51-S1.DOC]

|  |  |  |  |  |
| --- | --- | --- | --- | --- |
| Compound | Molecular weight (Da) | SRM transition  Parent *m/z* > Product *m/z* | Collision energy (v) | Authentic standard available for quantitation? |
|  |  |  |  |  |
|  |  |  |  |  |
| 1 (chloro-nicotinic acid) | 157 | 157.9 > 122.0 | 24 | ✓ |
| IMI-NH (guanidine) | 210 | 211.0 > 126.1 | 17 | 🗴 |
| IMI-urea (urea) | 211 | 212.0 > 128.1 | 24 | ✓ |
| IMI-NNH_2_ (hydrazone) | 225 | 226.0 > 126.0 | 22 | 🗴 |
| IMI-de | 229 | 230.1 > 110.9 | 22 | ✓ |
| IMI-NNO (nitrosoimine) | 239 | 240.0 > 209.0 | 22 | 🗴 |
| IMI-ole (olefin) | 253 | 254.1 > 205.7 | 17 | ✓ |
| IMI (imidacloprid) | 255 | 256.1 > 208.9 | 17 | ✓ |
| IMI-5-OH, IMI-4-OH (4/5-hydroxy) | 271 | 272.1 > 191.2 | 22 | ✓ (IMI-5-OH) |
| IMI-diol (4,5-dihydroxy) | 287 | 288.0 > 207.0 | 22 | 🗴 |
| IMI-5-conj, IMI-4-conj (sugar conjugates) | 433 | 434.0 > 272.0 | 22 | 🗴 |
|  |  |  |  |  |
