# Supplementary material for: Mutation of a nicotinic acetylcholine receptor β subunit is associated with resistance to neonicotinoid insecticides in the aphid Myzus persicae
Source: BMC Neurosci. 2011 May 31;12:51. doi: 10.1186/1471-2202-12-51 (PMC3121619; doi:10.1186/1471-2202-12-51)
Supplement: Additional file 3 — Fold change in expression of CYP6CY3 and additional P450 genes in the M. persicae clones FRC and 5191A (compared to the susceptible reference clone 4106A) as determined by quantitative PCR. For each EST the most similar Acyrthosiphon pisum P450 gene is detailed. [file 1471-2202-12-51-S3.DOC]

| **Gene/EST description** | **Est/gene ID** | **4106a** | **4106a**  **95% CL** | **FRC** | **FRC**  **95% CL** | **5191a** | **5191a**  **95% CL** |
| --- | --- | --- | --- | --- | --- | --- | --- |
| CYP 4 like (Ap CYP380C3) | 2519 | 1.00 | 0.18 | 2.84 | 0.88 | 0.18 | 0.03 |
| CYP 4 like (Ap CYP380C5) | 1504 | 1.01 | 0.23 | 2.61 | 0.45 | 0.45 | 0.05 |
| CYP6 like (Ap CYP6CY7) | 26873 | 1.02 | 0.24 | 2.36 | 0.28 | 1.17 | 0.32 |
| CYP6 like (Ap CYP6CY4) | 74544 | 1.00 | 0.11 | 7.24 | 2.19 | 7.68 | 0.75 |
| CYP6 like (Ap CYP6CY17) | 4886 | 1.06 | 0.49 | 3.09 | 0.39 | 1.01 | 0.26 |
| CYP6 like (Ap CYP6CY8) | 1501 | 1.01 | 0.20 | 2.40 | 0.04 | 0.78 | 0.49 |
| Mp CYP6CY3 | Mp CYP6CY3 | 1.03 | 0.37 | 28.03 | 7.28 | 22.04 | 1.80 |
