# Supplementary material for: Mutation of a nicotinic acetylcholine receptor β subunit is associated with resistance to neonicotinoid insecticides in the aphid Myzus persicae
Source: BMC Neurosci. 2011 May 31;12:51. doi: 10.1186/1471-2202-12-51 (PMC3121619; doi:10.1186/1471-2202-12-51)
Supplement: Additional file 4 — Sequences of primers used in this study. Primer sequences are listed along with the purpose for which they were used. [file 1471-2202-12-51-S4.DOC]

| **Name** | **Sequence** | **Purpose** |
| --- | --- | --- |
| MPa1F1 | GGTCAGCAACAACACCGACACC | PCR amplification of nAChRα1 |
| MPa1R1 | TCGGCAGGCAGATAGAAGACCA | PCR amplification of nAChRα1 |
| Mpa1F2 | CGACACCGTCCTGGTCAAGC | Sequencing of nAChRα1 |
| Mpa1R2 | GACCAGCACCGACAGGTAAG | Sequencing of nAChRα1 |
| Mpa2F1 | GCCAAACGGCTTTACGACGATC | PCR amplification of nAChRα1 |
| Mpa2R1 | GAACACCAGCACGGACAGGAAC | PCR amplification of nAChRα1 |
| Mpa2F2 | CGACGATCTGCTGAGCAACTAC | Sequencing of nAChRα1 |
| Mpa2R2 | GACAGGAACGATATACCCACGC | Sequencing of nAChRα1 |
| Mpa3F1 | CGGATGCAAAAAGGCTGTACGA | PCR amplification of nAChRα1 |
| Mpa3R1 | GGTAAAACACAAGTATCGTGAG | PCR amplification of nAChRα1 |
| Mpa3F2 | GGCTGTACGACGACCTTTTATC | Sequencing of nAChRα1 |
| Mpa3R2 | GAAACGATATCCCCATACAAGG | Sequencing of nAChRα1 |
| Mpa4F1 | GATGACCTTCTGAGCAACTAC | PCR amplification of nAChRα1 |
| Mpa4R1 | GTCAGTCCGACACAAGGGATG | PCR amplification of nAChRα1 |
| Mpa4F2 | CAACTACAATCGGCTGATCAG | Sequencing of nAChRα1 |
| Mpa4R2 | GTCCGACACAAGGGATGATC | Sequencing of nAChRα1 |
| Mpb1F1 | GTCCAGAACATGACCGAAAAAG | PCR amplification of AChRβ1 |
| Mpb1R1 | CGCACAGGAAAGATATAAGGAC | PCR amplification of AChRβ1 |
| Mpb1F2 | GACCGAAAAAGTCAATGTCCAG | Sequencing of AChRβ1 |
| Mpb1R2 | AAGATATAAGGACCGTGGGCAG | Sequencing of AChRβ1 |
| CYP6F | CGGGGTGACGATCATCTA TT | Q PCR (validation of microarray results for CYP6CY3) |
| CYP6R | GGGTGGTCTTTTGACAAA GC | Q PCR (validation of microarray results for CYP6CY3) |
| MpActF1 | ggtgtctcacacacagtgcc | Q PCR amplification of the actin reference gene |
| MpActR1 | cggcggtggtggtgaagctg | Q PCR amplification of the actin reference gene |
| Aph1R | TGG TAT ACA CGT TGG TTC TC | Q PCR amplification of the *para* reference gene |
| Aph18 | gaccacgagcttccccggtg | Q PCR amplification of the *para* reference gene |
| Mp1aceR3 | CACTGTAGAGCCATTAGCTG | Q PCR amplification of the *ace-1* reference gene |
| Mp1aceF4 | TAACGTAGTAGTGCCAAAGC | Q PCR amplification of the *ace-1* reference gene |
| 2519F2 | atgccatgttgtcgatgaaa | Q PCR (validation of microarray results for EST 2519) |
| 2519R2 | ctgtctcgtgatcgaatcgt | Q PCR (validation of microarray results for EST 2519) |
| 1504F | ggccgtgtaggatttggtta | Q PCR (validation of microarray results for EST 1504) |
| 1504R | gtacattcggcctttctgga | Q PCR (validation of microarray results for EST 1504) |
| 26873F | catattcagacgagcgacga | Q PCR (validation of microarray results for EST 26873) |
| 26873R | aagtatcgggatcggggtaa | Q PCR (validation of microarray results for EST 26873) |
| 74544F | tgaacaagcaaccaaagtagga | Q PCR (validation of microarray results for EST 74544) |
| 74544R | tgtccaaaatgacggacaaa | Q PCR (validation of microarray results for EST 74544) |
| 4886F | ttgccgaatcagaaatgaaa | Q PCR (validation of microarray results for EST 4886) |
| 4886R | atacaacgccgttctttggt | Q PCR (validation of microarray results for EST 4886) |
| 1501F2 | tgccaacttttggatggact | Q PCR (validation of microarray results for EST 1501) |
| 1501R2 | ggacgaacgtaaggcacatt | Q PCR (validation of microarray results for EST 1501) |
